# Safety of Hydroxychloroquine for COVID-19 Prophylaxis in Healthcare Workers: A Cross-Sectional Study

**DOI:** 10.7759/cureus.79660

**Published:** 2025-02-25

**Authors:** Kunj H, Pramila Yadav, Dhande M, Sayali Patil

**Affiliations:** 1 Department of Surgery, James Paget University Hospital, Norfolk, GBR; 2 Department of Pharmacology, D. Y. Patil University, Navi Mumbai, IND; 3 Department of Pharmacology, Mahatma Gandhi Memorial (MGM) Medical College and Hospital, Navi Mumbai, IND

**Keywords:** adverse effects, covid-19, hcq, healthcare professionals, healthcare workers, hydroxychloroquine, prophylaxis

## Abstract

Background: In March 2020, the World Health Organization (WHO) declared SARS-CoV-2 a pandemic after identifying it as the causative agent of COVID-19. Hydroxychloroquine (HCQ) was widely used as a prophylactic measure among healthcare workers (HCWs). However, its use as a preventive medication in asymptomatic individuals, particularly given its known adverse effect profile, remains relatively unexplored. This study aimed to evaluate the safety of HCQ prophylaxis among HCWs in Mumbai, India.

Materials and methods: A survey-based, cross-sectional study was conducted using a self-designed questionnaire distributed to HCWs in Mumbai who had direct or indirect patient contact and were taking HCQ for COVID-19 prevention.

Results: A total of 125 responses were collected, with males accounting for 80 (64%) of the participants. The majority of HCWs had a medical doctor background, and the mean age was 41.8 years. Thirty-eight HCWs (30.4%) reported frequent exposure to COVID-19, while 106 (84.8%) had direct patient contact. A minority (34 participants, 27.2%) received HCQ prophylaxis for seven weeks, and 123 (98.4%) adhered to the standard prophylactic regimen. Comorbidities were present in 27 (21.6%) HCWs, with hypertension (19, 15.2%) being the most common.

Among the participants, 64 (51.2%) underwent an electrocardiogram (ECG) before prophylaxis, while 56 (44.8%) had follow-up ECGs. Adverse effects were reported by 44 (35.2%) HCWs, with a statistically significant higher incidence among females. The most common adverse effects were gastrointestinal symptoms (48, 38.4%). While HCWs with hypertension and diabetes mellitus experienced more adverse effects, this association was not statistically significant. Cardiac adverse effects were reported in 13 cases (10.5%); however, no significant cardiovascular complications were observed.

Conclusions: This study found a higher occurrence of adverse effects among female participants, though previous research does not provide conclusive evidence for this finding. The incidence of gastrointestinal side effects was consistent with other studies on HCQ prophylaxis among HCWs. Although HCWs with hypertension and diabetes mellitus experienced more adverse effects, these were not statistically significant. No serious cardiovascular effects were observed. Given the evolving landscape of COVID-19 treatment and prevention, further large-scale trials are necessary to establish the safety and efficacy of HCQ prophylaxis among HCWs.

## Introduction

SARS-CoV-2 is the virus responsible for COVID-19. The first cases of COVID-19 were diagnosed in December 2019 in Wuhan, China. As the outbreak spread beyond Wuhan, the World Health Organization (WHO) declared COVID-19 a global pandemic on March 11, 2020. Nguyen et al. demonstrated that healthcare workers (HCWs) face a significant risk of SARS-CoV-2 infection due to their job-related exposure patterns [1]. Additionally, Liu et al. and Chan et al. indicated that HCWs primarily contract SARS-CoV-2 while working in hospital settings [2,3]. Huang et al. revealed that 80% of SARS-CoV-2 patients transmitted the virus to others even when asymptomatic. Because no proven antiviral treatment exists, SARS-CoV-2 spreads rapidly from person to person [4].

According to Ben-Zvi et al., hydroxychloroquine (HCQ) is a derivative used to treat rheumatoid arthritis, systemic lupus erythematosus, and malaria [5]. HCQ mitigates tissue damage and immune response by modulating immune cells, blocking harmful substances, and influencing cytokine activity [6]. HCQ may counteract SARS-CoV-2 by increasing lysosomal pH, thereby reducing intracellular iron levels. This process inhibits enzymes responsible for adding sugar molecules to viral particles, ultimately altering their glycosylation patterns [7].

Studies have analyzed SARS-CoV-2's response to HCQ. Yao et al. investigated HCQ's effects on viral entry and replication in infected Vero cells using physiologically based pharmacokinetic models [8]. Gautret et al., in an open-label, non-randomized trial, reported that HCQ reduced viral presence in 20 test subjects. Similarly, a study conducted in Wuhan, China, found that HCQ aided the recovery of 25 out of 31 patients [9,10]. Furthermore, Gautret et al. observed that combining HCQ with azithromycin further decreased SARS-CoV-2 respiratory viral load and indicated that HCQ reduced viral levels in patient samples within the first six days of treatment [10].

Recognizing the potential benefits of HCQ, the Indian Council of Medical Research (ICMR) recommended on March 22, 2020, that HCWs take HCQ prophylactically to prevent SARS-CoV-2 infection. Following a major outbreak, a South Korean nursing facility also administered HCQ to curb new infections [11]. A study aimed to determine whether similar strategies could be effective in countries like Italy, which faced a high burden of COVID-19 despite having an advanced healthcare system [12].

Previous studies assessing HCQ's adverse drug reactions primarily focused on symptomatic patients. However, data on its side effects in asymptomatic individuals remain limited. Key concerns include its interactions with other drugs and the appropriate dosage for prophylactic use. As an increasing number of asymptomatic individuals take HCQ as a preventive measure, an urgent need arises for studies evaluating its safety profile.

To address this gap, we conducted a survey to assess HCQ safety among HCWs in Mumbai. This study evaluated HCQ's adverse effects and examined their prevalence concerning demographic variables such as age, gender, and medical history. Additionally, it assessed HCWs' adherence to prophylactic protocols and the implementation of electrocardiogram (ECG) monitoring before and during HCQ use. Using a cross-sectional survey design, we aimed to identify potential safety risks associated with HCQ prophylaxis in asymptomatic individuals and determine its overall safety for frontline HCWs.

## Materials and methods

Study design

A cross-sectional survey was conducted to assess the safety of HCQ prophylaxis among HCWs in Mumbai, India. Data were collected over six months, from May 2021 to November 2021. A structured questionnaire was used to obtain information on the side effects of HCQ administration, as well as demographic details, HCQ prescription duration and dosage, and the frequency of ECG testing before and during the prophylaxis period. The questionnaire was validated by an expert, and a preliminary test was conducted before its final implementation. A convenience sampling method was employed. The Institutional Ethics Committee for Biomedical and Health Research of Dr. D. Y. Patil Medical College and Hospital approved the study (approval number: DYP/IECBH/2020/015, approval date: July 28, 2021).

Selection criteria

This survey included 125 HCWs taking HCQ as prophylaxis, all of whom had direct or indirect patient contact and provided informed consent to participate. The inclusion criteria required participants to be HCWs in Mumbai, India, who interacted with patients and actively used HCQ for COVID-19 prophylaxis. Only those who provided informed consent were eligible. The exclusion criteria encompassed individuals from the general population, HCWs not taking HCQ for prophylaxis, and those who did not consent.

Study tool

The questionnaire was electronically distributed via Google Forms (Google LLC, Mountain View, CA, USA), a web-based survey tool that allowed participants to access and complete it on computers or smartphones at their convenience. The study ensured voluntary participation, enabling HCWs to respond freely without external pressure.

Statistical analysis

Electronic data were exported from the database to Microsoft Excel spreadsheets (Microsoft Corporation, Redmond, WA, USA). Categorical data were presented as numbers and percentages. The analyzed data were displayed in an appropriate graphical format based on the data type. The study utilized Stata version 16 (StataCorp LLC, College Station, TX, USA) for statistical analysis. A forest plot was used to illustrate the impact of age, gender, and medical conditions on the risk of adverse outcomes.

## Results

A total of 125 HCWs participated in this cross-sectional survey study. The data revealed that 80 participants (64%) were male. The median age of the participants was 41.8 years, with 45 (36%) between the ages of 41 and 50. Additionally, 108 participants (86.4%) were doctors. The findings also showed that 38 HCWs (30.4%) had regular contact with COVID-19 patients, while 106 HCWs (84.8%) had direct patient contact with individuals who tested positive for COVID-19 (Table 1).

**Table 1:** Demographic profile HCW: healthcare worker, COVID-19: coronavirus disease 2019, ECG: electrocardiogram

Demographics	Participants n=125 (%)
Mean age	41.8 years
Median age	42 years
Sex	
Male	80 (64)
Female	45 (36)
HCW category	
Doctor	108 (86.4)
Nurse	14 (11.2)
Others	3 (2.4)
Direct contact with COVID-19 patients	
Yes	106 (84.8)
No	19 (15.2)
COVID-19 test	
Positive	12 (9.6)
Negative	113 (90.4)
Comorbidities	
Hypertension	19 (15.2)
Ischemic heart disease	3 (2.4)
Diabetes mellitus	3 (2.4)
Respiratory disorder/asthma	5 (4)
Hypothyroidism	1 (0.8)
Obesity	1 (0.8)
ECG before HCQ	
Yes	56 (44.8)
No	69 (55.2)
Follow-up ECGs done	
Yes	64 (51.2)
No	61 (48.8)
Duration of prophylaxis in weeks	
1	4 (3.2)
2	0 (0)
3	3 (2.4)
4	10 (8)
5	5 (4)
6	9 (7.2)
7	34 (27.2)
≥7	60 (48)
Frequency of exposure	
Regularly	38 (30.4)
Sometimes	79 (63.2)
Not answered	8 (6.4)
Inquiry made before starting HCQ prophylaxis	
Allergy	24 (19.2)
G-6-PD deficiency	18 (14.4)
Arrhythmias	60 (48)
Medication	26 (20.8)
Retinopathy	12 (10.4)
None	60 (48)

The duration of HCQ prophylaxis ranged from one to 14 weeks, with a maximum of 34 participants (27.2%) taking it for seven weeks. Among those who followed the standard dosage regimen, 11 participants (8.8%) tested positive for COVID-19. Additionally, 27 participants (21.6%) had comorbidities, with hypertension being the most prevalent (19 participants, 15.2%). The vast majority of the study population (123 participants, 98.4%) adhered to the standard prophylaxis regimen, while only two participants (1.6%) followed a modified regimen. Approximately 64 participants (51.2%) underwent an ECG before initiating prophylaxis, whereas only 56 participants (44.8%) had follow-up ECGs.

**Figure 1:**
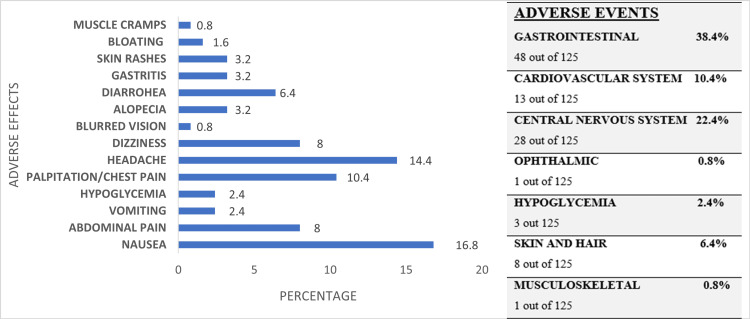
Distribution of adverse effect profile

The frequency and distribution of adverse effects are presented in Figure 1. A total of 44 participants (35.2%) reported experiencing 102 adverse effects, while 81 participants (64.8%) reported none. Among hypertensive participants, 10 individuals (8%) experienced a higher incidence of adverse effects, with four participants (3.2%) reporting palpitations and chest pain.

**Figure 2:**
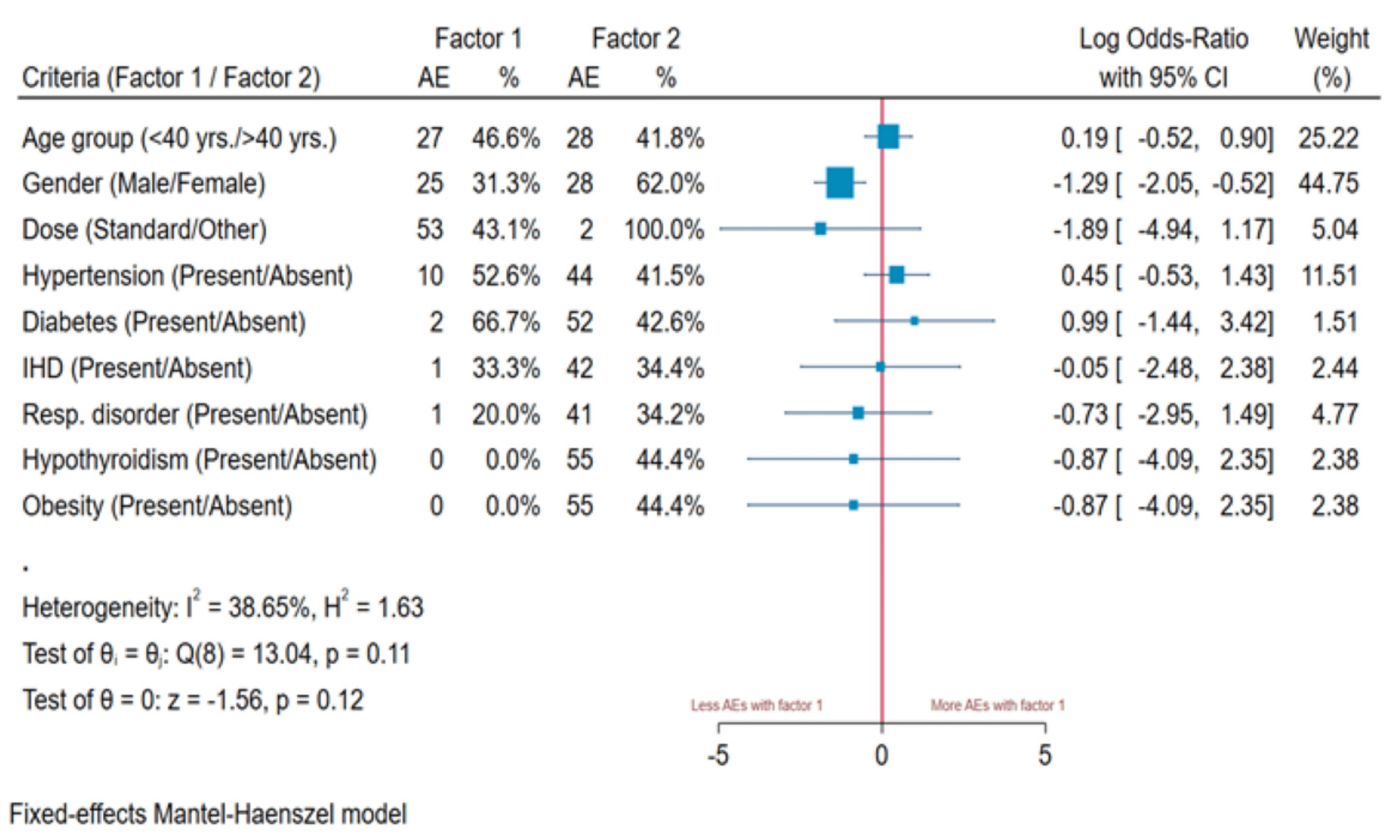
OR of adverse effects associated with age, gender, and comorbidities AE: adverse effects, OR: odds ratio, CI: confidence interval, IHD: ischemic heart disease

Figure 2 displays the odds ratio (OR) and 95% confidence interval (CI) for factors associated with the probability of adverse medication effects from HCQ preventive therapy, presented on a logarithmic scale. The ORs for the risk of adverse effects related to age, gender, and comorbidities, including hypertension, diabetes mellitus, hypothyroidism, obesity, respiratory disorders, and ischemic heart disease. A statistically significant increased risk of adverse effects was observed in females (OR: -1.29, 95% CI: -2.05, -0.52), while associations with all other factors remained weak.

## Discussion

Most anti-COVID-19 medications, including remdesivir, lopinavir, ribavirin, molnupiravir, HCQ, azithromycin, chloroquine, tocilizumab, and ivermectin, either failed to provide significant benefits to patients or only slightly reduced the risk of death [12,13]. According to Rajasingham et al. and Naggie et al., HCQ, a chloroquine derivative, was tested due to its presumed ability to combat viral infections, including COVID-19 [14,15].

India faced challenges in determining whether HCQ should be used for COVID-19 prevention after it was found ineffective in treating hospitalized patients. Similar conclusions were drawn by Rojas-Serrano et al. and McKinnon et al. [16,17]. In March 2020, the ICMR recommended HCQ for HCWs treating COVID-19 patients and asymptomatic household members of confirmed cases. A study from that period suggested HCQ had some effectiveness.

Placebo-controlled trials were conducted to assess HCQ's post-exposure prophylaxis (PEP) benefits for HCWs. However, these trials showed only minor advantages and required larger study populations. Subsequent trials evaluating HCQ’s ability to protect HCWs from COVID-19 encountered similar limitations [11-15,18].

Studies have yielded mixed results regarding HCQ's effectiveness in preventing COVID-19 transmission after exposure to SARS-CoV-2 [19]. Boulware et al. found that HCQ did not effectively prevent COVID-19 transmission post-exposure [14]. While HCQ was considered for both prevention and treatment, most studies relied on database findings. India required more comprehensive national trials to evaluate chloroquine’s effectiveness as a PEP treatment for COVID-19.

During this period, a medical team monitored 125 HCWs who took HCQ as a preventive measure to assess its safety. Most participants (86.4%) were doctors, with an average age of 41.8 years. The revised ICMR strategies categorized HCQ prophylaxis personnel into two groups. Category 1 personnel were required to take 400 mg of HCQ twice on the first day, followed by a weekly dose for three weeks during meals. Category 2 included HCWs and frontline workers interacting with COVID-19 patients or working in non-COVID-19 areas.

To comply with ICMR guidelines, individuals in Category 2 undergoing HCQ prophylaxis had to undergo ECG testing, especially if they developed cardiovascular symptoms such as chest pain. ECG tests were used to assess the QT interval before initiating HCQ treatment and during extended prophylaxis beyond eight weeks [20].

Participants took HCQ prophylaxis for either one or 14 weeks. Among them, 34 (27.2%) received treatment for seven weeks, while 60 (48%) continued beyond seven weeks following study guidelines. The team adhered to current HCQ guidelines when making these decisions. Hong et al. reviewed 10 medical trials, noting variations in follow-up periods ranging from 28 to 180 days. Research teams tested different HCQ dosage regimens, including daily doses from 200 to 600 mg, additional loading doses, and weekly or three-week plans, with each study implementing a unique treatment duration [21].

Among those who followed the standard dose regimen, only 11 (8.8%) tested positive for COVID-19. However, this study found that extended HCQ prophylaxis did not significantly reduce SARS-CoV-2 infection. Despite its limitations, the findings suggest that HCQ may have provided some protection against COVID-19, though the duration of prophylaxis did not appear to influence this outcome.

HCQ has been deemed safe for patients with autoimmune diseases for many years [15,16]. Its reported risk levels are similar to those observed in rheumatological conditions such as systemic lupus erythematosus. However, its effects over extended treatment periods remain under study [22]. When administered at the correct dosage, HCQ is considered safe for short-term malaria treatment [5].

Among the 125 HCWs taking HCQ, 44 (35.2%) reported 102 adverse effects, primarily gastrointestinal issues (48 cases, 38.4%). A high incidence of gastrointestinal side effects has been noted when HCQ is used to treat COVID-19 patients [23,24]. This study confirmed previously documented HCQ side effects [25,26].

The OR for adverse effects in relation to age, gender, and comorbidities, such as hypertension, diabetes mellitus, hypothyroidism, obesity, respiratory disorders, and ischemic heart disease, showed a statistically significant association with female patients. However, associations with other factors were weak. Female participants exhibited a higher probability of adverse effects, though no plausible correlation or explanation for this finding could be identified [26-28].

Of the 65 HCWs who took HCQ prophylaxis for seven weeks, 13.6% reported adverse effects. Additionally, 19 (15.2%) HCWs who took HCQ for more than seven weeks experienced adverse effects. However, no statistically significant correlation was found. Among 19 hypertensive participants, four (3.2%) experienced chest pain and palpitations. The Mantel-Haenszel model suggested a modest relationship with other parameters. One possible explanation for this could be the study's limited sample size.

Limitations of the study

This study has several limitations, including a small sample size, the absence of control participants, and a lack of follow-up data. Additionally, it relies on participant recall and does not assess the severity of side effects. A larger sample size, including more participants who did not adhere to the standard regimen, would have allowed for a more comprehensive analysis. The study also did not investigate pharmaceutical interactions, particularly with azithromycin, which has been associated with QTc interval prolongation and cardiac arrhythmias. Due to major design flaws, research conducted during the outbreak failed to yield definitive results. The study’s power was limited as it did not meet its planned participant recruitment target. As a result, this trial, like many others, has not conclusively determined HCQ’s efficacy.

Despite its limitations, this study provides valuable field-based information on HCQ safety for HCWs. However, further research opportunities remain. The study’s limited participant pool, only 125 HCWs, suggests the need for larger-scale research across multiple locations. The absence of a control group restricts comparative analysis, emphasizing the need for randomized controlled trials to establish definitive outcomes. Longitudinal studies are necessary to investigate long-term safety, as no follow-up data were obtained. Additional research is required to understand why female HCWs experienced more adverse effects. Although this study contributes useful insights, it underscores the need for broader investigations into HCQ prophylaxis among HCWs.

## Conclusions

The study demonstrated a higher incidence of adverse reactions among female participants but did not provide conclusive evidence based on previous studies. This study's findings align with prior research on HCQ prophylaxis among HCWs, as gastrointestinal adverse effects were among the most commonly reported side effects. However, the additional adverse effects reported by HCWs with hypertension and diabetes mellitus did not reach statistical significance. The assessment confirmed HCQ’s favorable cardiac safety profile for this specific group of HCWs.

Research on COVID-19, both clinical and observational, began soon after the pandemic started. However, many trials were prematurely terminated due to evolving perspectives on HCQ’s effectiveness and advancements in vaccine development. Despite various research limitations, this study successfully provided valuable insights into the safety profile of HCQ prophylaxis for HCWs. Nevertheless, long-term safety and efficacy assessments of HCQ as a prophylactic treatment require further large-scale, controlled trials.
